# Pathophysiology of Coronary Collaterals#

**DOI:** 10.2174/1573403X113099990005

**Published:** 2014-02

**Authors:** Michael Stoller, Christian Seiler

**Affiliations:** Department of Cardiology, University Hospital, Bern, Switzerland

**Keywords:** Coronary circulation, collateral circulation, physiopathology, myocardial ischemia.

## Abstract

While the existence of structural adaptation of coronary anastomoses is undisputed, the potential of coronary collaterals to be capable of functional adaptation has been questioned. For many years, collateral vessels were thought to be rigid tubes allowing only limited blood flow governed by the pressure gradient across them. This concept was consistent with the notion that although collaterals could provide adequate blood flow to maintain resting levels, they would be unable to increase blood flow sufficiently in situations of increased myocardial oxygen demand.

However, more recent studies have demonstrated the capability of the collateral circulation to deliver sufficient blood flow even during exertion or pharmacologic stress. Moreover, it has been shown that increases in collateral flow could be attributed directly to collateral vasomotion.

This review summarizes the pathophysiology of the coronary collateral circulation, ie the functional adapation of coronary collaterals to acute alterations in the coronary circulation.

## INTRODUCTION

While the existence of structural adaptation of coronary anastomoses is undisputed, their capability of functional adaptation has been questioned. For many years, collateral vessels were thought to be rigid tubes, allowing only limited blood flow, governed by the pressure gradient across them. This concept was consistent with the notion that although collaterals could provide adequate blood flow to maintain resting levels, they would be unable to increase blood flow sufficiently in situations of increased myocardial oxygen demand.

However, more recent studies, mainly in animal models, but also in humans, have demonstrated functional collateral adaptation to deliver sufficient blood flow even during exertion or pharmacologic stress. Moreover, it has been shown that increases in coronary collateral flow could be attributed directly to collateral vasomotion. It is, therefore, necessary to differentiate between changes in collateral flow due to resistance changes in collateral vessels and in coronary resistance vessels.

Earlier reviews on coronary hemodynamics [1-6] have provided information on the coronary circulation and this knowledge has added to gain understanding in the sometimes heterogenous response of collateral flow. The comprehensive work by Seiler [7] presents the most current review on the coronary collateral circulation while other reviews focusing on the topic are either outdated [8], have interpreted pathophysiology in the sense of chronic structural rather than functional adaptation [9], or have focused on collateral flow changes during exercise [10]. This review concentrates on the functional adapation of coronary collaterals to acute, as opposed to the chronic adaptations, ie arteriogenesis.

The aim of this review is to show that collateral function exists and to present the salient features relating to the functional adaptation of coronary collaterals. We start with the role of flow-mediated dilation, first by the concerted effect of exercise, then by pharmacologic stress with adenosine. The observed heterogenous responses produced by vascular adaptations in both coronary collateral and coronary non-collateral vessels will then lead us to examine the consequences of a heterogeneous vascular resistance distribution on coronary blood flow. Subsequently, we review changes of collateral vasomotion to stimuli other than those involved in fluid shear stress mediated collateral function. Last, influences on collateral function outside of the coronary circulation, ie extracoronary determinants will be discussed.

## FLUID SHEAR STRESS AND VASOMOTOR FUNCTION

The calibre of conduit arteries is regulated by tangential fluid wall shear stress, modifying the endothelium-dependent production of vasoactive mediators and consequently determining vascular tone. An increase in coronary flow is accompanied by an increase in wall shear stress consequently leading to vasodilation a phenomenon coined flow-mediated vasodilation. The functional vasodilator response taking place in the acute, as opposed to the chronic setting, can be seen as a limited shear stress control to prevent coronary blood flow rate from causing endothelial damage during a short-term, acute maximum flow state [11-13].

In the chronic setting, shear stress levels are regulated to a steady state, ie constant shear stress regulation by a change in structure as opposed to a change in function in the acute setting. Thus, the functional changes become more permanent structural modifications through a remodeling process. This response has been termed chronic adaptive and relates to the process of arteriogenesis in general, and to the process of coronary collateral growth in particular.

### Exercise-induced Coronary Collateral Function

The influence of exercise on collateral blood flow is highly relevant for several reasons. First of all, exercise is the most important physiological stimulus for increased myocardial oxygen demands. Second, the results are applicable in the clinical setting and consequently of high clinical relevance. Third, exercise acts in concert on the coronary circulation, which allows to observe the net effect of several influencing factors on collateral flow and vasomotion.

#### Experimental Studies

Experimental studies in the canine collateral circulation have typically made use of the ameroid constrictor model. The hygroscopic ameroid material absorbs water and progressively occludes the arterial lumen, leading to total occlusion in approximately 2½ weeks. The gradual occlusion practically prevents the occurrence of myocardial infarction, as the pre-existing collateral vessels are given sufficient time to develop. By 4-6 weeks after gradual occlusion, blood-flow to the collateral-dependent area is normal both in volume and transmural distribution and by 6 months the transformation process appears to be completed [8].

Thus, Lambert *et al*., examining collateral blood flow in dogs, a minimum of 6 months after placement of an ameroid constrictor of the left circumflex artery, studied the mature intercoronary collateral circulation [14]. Myocardial blood flow increased three-fold in the collateralized region during exercise and was not different from normally perfused control regions. Furthermore, the transmural distribution was uniform at rest and during exercise in both the control and collateral-dependent regions.

**Fig. (1):**
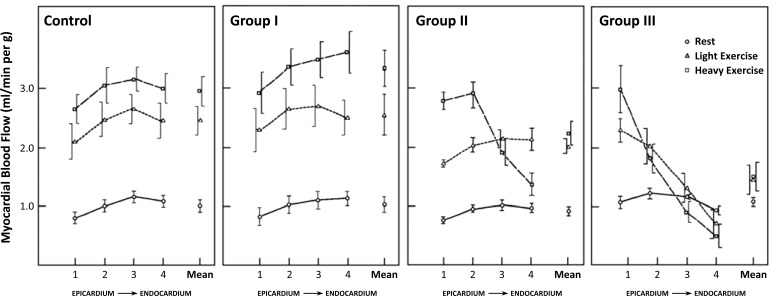
Myocardial blood flow in the collateral-dependent myocardial region of 14 dogs after 1 month after placement of an ameroid constrictor of the left circumflex artery (group I to III) and in 7 normal dogs (control). Data (means ± SE) are shown for 4 transmural layers from epicardium to endocardium at rest and during two levels of treadmill exercise. Group I shows collateralization sufficient to allow completely normal increase in myocardial blood flow during both levels of exercise. Group II shows subnormal increase in blood flow during light exercise and relative underperfusion during heavy exercise, expressing as transmural redistribution of flow towards the subepicardium. Group III shows collateralization sufficient only at rest, with already light exercise leading to redistribution towards the epicardium and heavy exercise leading to an actual decrease of myocardial blood levels in the inner layers to below resting levels (transmural steal). Dot inside symbol denotes significant differences from corresponding control (p<0.05). [Data from Bache and Schwartz [15]].

Bache *et al*., on the other hand, studied the collateral blood flow in dogs well before the development of a fully mature collateral system [15]. Collateral blood flow was determined at rest and during two levels of exercise one month after placement of an ameroid constrictor on either the left circumflex or left anterior descending artery. Myocardial blood flow at rest in the collateral-dependent area was normal in all dogs. However, during exercise, three different patterns of response occurred during treadmill exercise in the animals with a collateral-dependent myocardial region (see Fig. **1**). Dogs in group I had blood flow similar to the normal group without occlusion at both exercise levels. Group II had normal blood flow during light exercise, but during heavy exercise a transmural redistribution of perfusion occurred in the collateralized area to result in subendocardial ischemia. In Group III, even light exercise resulted in subendocardial ischemia and heavy exercise resulted in an actual decrease below resting levels, ie transmural steal.

The differential responses as illustrated by Bache *et al*. exemplify the response of collateral blood flow in dependence of the degree of collateral maturity also found in other studies (see Fig. **1**) [16-19]. Underdeveloped collaterals are able to satisfy normal basal flow needs, but they demonstrate partly limited capacity when demands are increased.

However, the studies in the chronic occlusion model could not differentiate whether increased collateral flow was due to changes of vascular tone in resistance vessels in the collateral-dependent myocardium alone or indeed partly attributable to active collateral vasomotion.

### Clinical Studies

**Chronic occlusion model. **Noninvasive techniques often employed in earlier clinical studies directed at the assessment of the functional capacity of coronary collaterals under exercise have, ipso facto, required the presence of a chronic total occlusion [20]. The chronic occlusion model, however, introduces a substantial selection bias, as only very well-developed collaterals, not representative for a majority of patients with chronic stable CAD, are included [21].

While modern myocardial perfusion imaging using positron emission tomography is able to measure actual perfusion, the only available studies investigating collateral function in response to exercise have employed thallium-201 perfusion imaging providing only a dichotomous measure of absent or present perfusion defects [22-24].

**Fig. (2):**
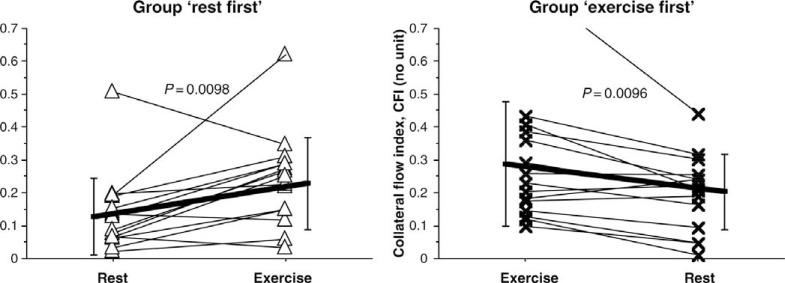
Individual changes (thin lines) of collateral flow index (collateral flow index; vertical axes) from the resting condition to the peak supine bicycle exercise condition in the group ‘rest first’ (left panel; triangular symbols) and vice versa in the group ‘exercise first’ (right panel; cross symbols). CFI increased during exercise in 24 patients and decreased in six patients (coronary steal). Thick lines indicate the mean CFI change between the resting and exercise condition. Error bars denote standard deviation. See text for further explanation. [Data from Togni *et al.* [25]].

**Acute occlusion model.** Togni *et al.* performed the first clinical study on coronary collateral behavior during isometric physical exercise [25]. 30 patients with chronic stable CAD underwent coronary balloon occlusion with collateral flow measurement before and during the last minute of a 6 min protocol of supine bicycle exercise during radial artery access coronary angiography. In these patients with non-occlusive CAD, collateral flow index [26] (CFI) instantaneously doubled during exercise as compared to the resting state (see Fig. **2**). Importantly, the last 10 patients underwent myocardial contrast echocardiography in addition to CFI measurements, which allowed determination of collateral resistance. It could be shown that collateral resistance decreased during exercise as compared to the resting state, to prove the concept that increasing collateral flow during exercise is at least partly attributable to direct collateral vasomotor function, ie collateral dilation. In agreement with the observations from the experimental studies, CFI decreased in a minority (a fifth) of patients during exercise.

### Adenosine and Flow-mediated Coronary Collateral Function

#### Exercise- vs Adenosine-induced Flow-mediated Vasodilation

Adenosine is a widely employed pharmacological agent for stress testing. As a metabolic vasodilator, adenosine has a predominant effect on arterioles <100 µm, [27, 28] with only weak dilator properties on arterial vessels >100 μm. Similarly, dipyridamole acts by increasing intravascular adenosine levels by inhibition of reuptake and deamination of adenosine. Exogenous adenosine produces profound vasodilation in the coronary microvasculature and causes increased flow with augmented fluid shear stress in upstream epicardial conductance arteries, which allows to examine the influence of the consequently incited flow-mediated vasodilation on coronary collateral function.

While both exercise and adenosine act principally by inducing flow-mediated vasodilation, the differential effects on coronary collateral function changes have to be considered. Most importantly of all, exercise encompasses a considerably more complex interplay of possible influencing factors than administration of a pharmacological agent, such as β-adrenergic activity [29-32], vasodilator influences by endothelium derived NO and increasing heart rate. As shown experimentally, coronary dilatation secondary to an increase in myocardial oxygen consumption does not produce identical changes in the distribution of coronary microvascular resistance as adenosine, especially when dilatation in smaller arterial microvessels is considered [27].

Furthermore, adenosine plays a significant role in ischemic coronary vasodilation [33, 34]. In the context that assessment of collateral function potentially induces ischemia as well, this fact requires consideration as any potential influence by exogenous adenosine might be attenuated.

#### Clinical Studies

**Chronic occlusion model.** McFalls *et al*. used positron emission tomography in 5 patients with chronic total occlusion of a major coronary artery to determine coronary flow reserve in entirely collateral-dependent myocardium in response to dipyridamole [35]. Dipyridamole acts via increased extracellular levels of adenosine through inhibition of the enzyme adenosine deaminase, which normally inactivates adenosine. During resting conditions, myocardial blood flow in the control group was 0.86±0.10 ml/g/min and in the patient group was 0.99±0.10 ml/g/min in normally perfused myocardium and 0.86±0.14 ml/g/min in collateral-dependent myocardium. Absolute coronary flow reserve in the control group was 4.1±0.8 and 3.1±1.1 in the collateral-independent normal regions of patients and 1.9±1.0 (p< 0.001) in patients with collateral-dependent regions.

Similarly, Vanoverschelde *et al*. performed PET to measure absolute regional myocardial blood flow in 26 anginal patients with chronic occlusion of a major coronary artery, but without previous infarction. Patients were separated according to normal or abnormal resting regional wall motion of the collateral-dependent myocardial region [36]. While myocardial blood flow at rest was similar among collateral-dependent segments of patients with and without segmental dysfunction, after intravenous dipyridamole, collateral-dependent myocardial blood flow increased by 205% in three patients with normal wall motion (from 0.78±0.05 to 2.38±0.54 ml/g/min) and by only 27% in eight patients with regional dysfunction (from 0.88±0.17 to only 1.12±0.44 ml/g/min).

**Acute occlusion model.** Piek *et al.* examined 38 patients with one-vessel CAD in an acute balloon occlusion model [37]. Arguing that the systemic effects of intravenously administrated vasodilators might have caused alterations in preload or afterload and thus have indirectly caused the enhanced collateral flow observed in prior studies, adenosine was given as intracoronary bolus in the presumable donor coronary artery while determining measures of collateral flow during a brief occlusion of the collateral-receiving artery. Patients were analyzed according to spontaneously visible or recruitable collaterals. Collateral blood flow in patients with only recruitable collaterals remained unchanged in response to adenosine. Conversely, in patients with spontaneously visible collaterals, collateral blood flow increased by 35% and transcollateral resistance decreased by 16.5%, indicating a collateral vasodilator response to adenosine. The intracoronary route of adenosine administration set the group with no spontaneously visible collaterals at a significant disadvantage and could possibly have explained the fact that no response of coronary collateral function could be induced by adenosine in this group.

**Fig. (3):**
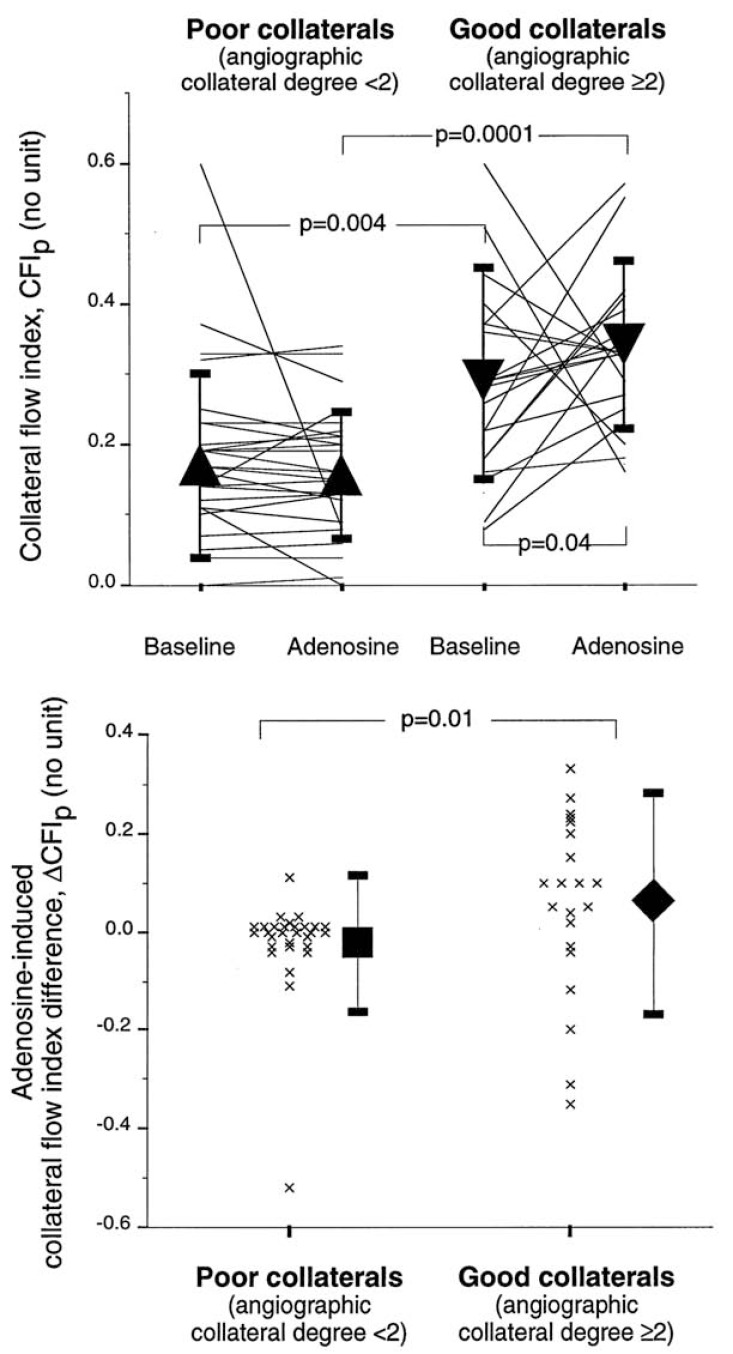
Upper panel: Individual, collateral flow index values at baseline and during intravenous adenosine infusion for patients with poor and good collaterals. The triangles indicate mean values (± standard deviation). Bottom panel: Individual, absolute CFIp changes in response to adenosine (i.e. CFIp during hyperemia − CFIp at rest) for the groups with poor and good collaterals, respectively. CFI remained practically unchanged during adenosine infusion in the group with poor collaterals. In the group with good collaterals, mean CFI increased significantly during adenosine infusion, but the response was highly variable. CFIp = pressure derived collateral flow index. See text for further explanation [Data from Seiler *et al.* [38]].

Therefore, Seiler *et al*. performed a similar study in 50 patients with CAD, again dividing patients in a group with and without spontaneously visible collaterals [38]. In contrast to the study by Piek *et al*., adenosine was given intravenously (140 µg/kg/min) for induction of flow-mediated vasodilation. In the absence of spontaneously visible collaterals, pressure-derived CFI remained practically unchanged under adenosine, but increased by a factor of 1.21 in the presence of spontaneously visible collaterals (see Fig. **3**). Of note, the response to adenosine in the group with good collaterals was highly variable, with CFI changes ranging from -0.3 to 0.3. Furthermore, 6 of 21 patients in this group showed a decrease in CFI under adenosine.

Similarly, Perera *et al*. performed a study in 33 patients with one-vessel CAD with coronary pressure-derived collateral assessment at rest and during intravenous adenosine (140 µg/kg/min) and found no change in CFI among patients with poorly developed collaterals, but an overall decrease during hyperemia with increasing collateral flow at rest [39].

Werner *et al*. assessed the functional reserve of collaterals in 62 patients with chronic total coronary occlusions without prior myocardial infarction [40]. In response to intravenous adenosine infusion (140 µg/kg/min), the collateral flow reserve was 1.15 ± 0.58, with 7% of patients having a value >2.0. Furthermore, in a third of patients, collateral flow decreased during pharmacological stress with adenosine.

### Summary

Collateral blood flow is able to increase in response to flow-mediated vasodilation induced by exercise or by administration of a pharmacological stress agent such as adenosine or dipyridamole. Furthermore, it has been demonstrated that direct collateral vasodilation is a contributing mechanism leading to the increase in collateral blood flow. Overall, coronary collaterals are able to meet increased demands of dependent myocardium as a function of their level of expression.

The observations of an absent increase in collateral flow in response to adenosine, when collateral vessels are not angiographically visible, seems consistent. Yet, this observation could be related to the acute occlusion model employed in assessing collateral blood flow. Coronary occlusion is likely to induce ischemia in the absence of good collaterals, which leads to ischemic vasodilation via release of endogenous adenosine. Typically and as exemplified in the study by Seiler *et al*. (see Fig. **3**) [38], angiographically visible collaterals have a CFI in a range, where they are likely to prevent ischemia during a brief occlusion. Conversely, recruitable collaterals typically have collateral flow insufficient to prevent ischemia. Therefore, ischemic (pre)dilation might preclude further vasodilation of poor collaterals by exogenous adenosine. However, this is in contrast to observations in both experimental [28], as well as in clinical studies [41-43], showing that even during coronary hypoperfusion there is substantial vasodilator reserve in the terminal vascular bed recruitable with arterioral vasodilators such as adenosine. However, adenosine might fail to enhance flow to a (severely) ischemic area even if it causes further vasodilation. This might be due to failure of adenosine to oppose small arteriolar vasoconstriction in hypoperfused myocardium [44, 45]. 

The decreases in collateral blood flow show the need for integration of further explanatory concepts, as outlined in the next section.

## VASCULAR RESISTANCE DISTRIBUTION

While a well-developed coronary collateral circulation often maintains adequate perfusion of dependent myocardium at rest, there are negative aspects, which manifest preferentially in the presence of a well-developed collateralization. As alluded to in the previous section, steal phenomena can occur during exercise and pharmacological stress testing, precipitating ischemia in a collateral-dependent myocardium.

Furthermore, alternative flow provided by collaterals, having well developed during constriction of an epicardial vessel, can be competitive with restored antegrade flow after coronary intervention. Evidence suggests that high functional flow may be a risk factor for restenosis.

The mentioned negative aspects of collateral circulation are all related to the (altered) distribution of vascular resistance, both at the level of microvasculature and at the level of the epicardial arteries. After explanation of the underlying biophysical mechanisms, these aspects will be discussed.

### Redistribution of Blood Due to Altering Microvascular Resistances

#### Biophysical Mechanisms of Coronary Steal

In normal myocardium, increases in contractile function are associated with increased metabolism and the increased metabolic demands are met predominantly by increased myocardial blood flow (and by increased oxygen extraction to a lesser extent) [1, 46]. Increased myocardial blood flow is achieved by metabolic coronary vasodilation.

In contrast to the spatially well orchestrated myocardial blood flow in the normal coronary circulation, coronary autoregulation in patients with CAD is disturbed. In the presence of a flow-limiting epicardial stenosis, autoregulation aims to keep blood flow constant by reducing the microvascular resistance in the dependent myocardium. In principle, this leads to an uneven distribution of microvascular resistance between different coronary territories.

**Fig. (4):**
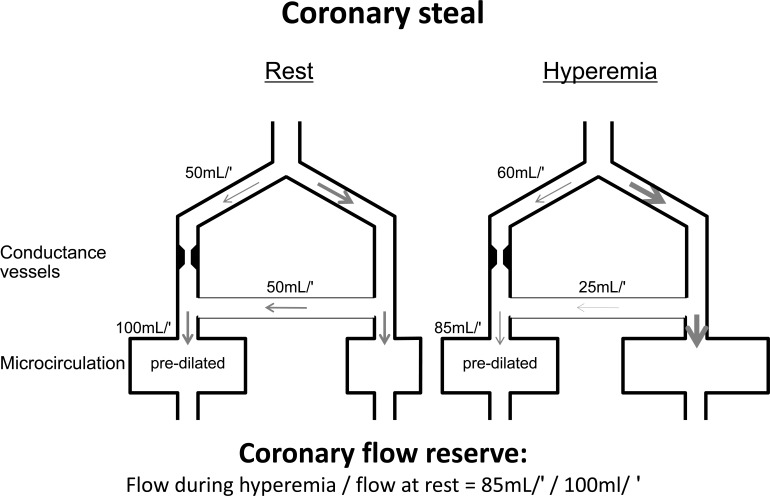
Diagram depicting coronary collateral steal. At rest, microcirculation of myocardial region distal to stenotic conductance vessel maintains normal perfusion by compensatory predilatation. In situation illustrated, half of perfusion (50 mL/min [mL/′]) is provided by antegrade flow through stenotic vessel and half by collaterals (50 mL/min) from contralateral nonstenotic vessel. During hyperemia, capacity for further arteriolar dilatation in poststenotic region (left) is exhausted, whereas it is intact on contralateral collateral-supplying side. Thus, microvascular resistance is more reduced on collateral-providing side than on poststenotic side, thereby reducing flow via collaterals (to 25 mL/min in this example) and causing net myocardial perfusion during hyperemia to be less (ie, 85mL/min) than at resting conditions (ie, 100mL/min). [Data from Seiler *et al.* [56]].

The amount of blood flow to a coronary territory affected by a flow-limiting epicardial stenosis is dependent on the antegrade flow through the stenosis and the collateral inflow from adjacent regions (see Fig. **4**). To compensate for the pressure drop across the stenosis, autoregulation aims to reduce microvascular resistance in the dependent myocardium accordingly. Collateral inflow depends on the pressure gradient between the origin of the donor vessel and the entry into the recipient vessel. When the dilator reserve of the recipient vessel is exhausted, flow becomes pressure-dependent. Antegrade flow through the stenosis is at its maximum and collateral inflow dependent on the perfusion pressure of the collateral-supplying donor vessel. Any reduction in the microvasular resistance of the collateral-supplying donor vessel by metabolic demand or dilator agents will thus lead to a decrease in the pressure gradient driving collateral flow. Net flow is consequently reduced and ischemia precipitated. The ensuing phenomenon of decreasing flow to a myocardial region in need during hyperemia below the resting level of flow is called coronary steal [47].

The before-mentioned redistribution of coronary flow due to altered microvascular resistances is further compounded in the setting of increased heart rate. Tachycardia increases myocardial oxygen demand per time and additionally by a contractile force-frequency effect [48]. Conversely, tachycardia shortens diastole and thus the time interval, in which almost all of the coronary blood flow occurs [49]. Reduced diastolic duration is normally adequately compensated for by metabolic vasodilation, matching the increased oxygen demand. However, in the presence of a flow-limiting stenosis, there is already autoregulatory predilatation to a certain degree. As a consequence, the capacity of further vasodilation and thus perfusion is limited. Beyond a critical heart rate, governed by the severity of the stenosis, hypoperfusion precipitates ischemia. A vicious cycle is incited, where increasing transmural tension due to the ischemia leads to actually heightened microvascular resistance further lowering perfusion. Additionally, increasing heart rate also increases the hemodynamic severity of a coronary stenosis due to increased turbulence, which further compromises coronary inflow [50]. 

Whereas coronary steal relates to the redistribution of flow between different coronary territories, there exists a similar situation between the outer and inner layers of the myocardium. In the case of coronary, ie ‘horizontal’ steal, the dilator reserve in the normal, or less stenotic adjacent myocardium is still preserved, when the dilator reserve in the post-stenotic region is already exhausted, and further vasodilation in the normal myocardium causes coronary steal by decreasing the pressure gradient driving the collateral flow. Analogously, in transmural, ie ‘vertical’ steal, the subepicardium still has preserved dilator reserve, when the subendocardium, exposed to higher extravascular compressive forces, has not [3, 51]. Redistribution from the subendocardium to the subepicardium will thus ensue similarly when there is further vasodilation in the epicardium.

#### Evidence for the Occurrence of Coronary Steal

Rowe, in 1970, was among the first to recognize the phenomenon of collateral steal [47]. Since then, several experimental studies have examined coronary steal [52-54]. Later, coronary steal was demonstrated also invasively [55-60], by PET [61] and modeled theoretically [62]. The clinical relevance of coronary steal depends on the prevalence of the phenomenon, which has been reported variably.

Invasive determination of the prevalence of collateral steal showed that occurrence was more frequent in patients with a chronic total occlusion. While in a patient population with non-occlusive CAD, the prevalence was 10% [56], in patients with chronic total occlusion it was observed in 46% [59], and in a third of 62 patients in another study [40]. 

In 1100 cardiac PET studies of patients with collateralized, occluded territories, 7% revealed coronary steal [63], whereas in a much smaller study using ammonia PET in 18 patients with multivessel CAD (10 with a CTO) [64], 8 patients (44%) showed a decrease in the perfusion of the collateralized region induced by stress-testing with dipyridamole.

Clinical suspicion can be raised by the capability of vasodilators to provoke or worsen ischemic symptoms [65, 66]. Furthermore, the nifedipine-induced exertional ECG ST segment depression or worsening of angina symptoms can also be used to give an estimate of the prevalence of collateral steal. Estimates based on these observations range from 10 to 20% [66-68]. 

#### Conditions for Coronary Steal

From the theoretic considerations at the beginning of this section it becomes clear that for coronary steal to occur, the following unequivocal and minimal conditions must be satisfied, in order of importance:

1. The dilator capacity of the collateral-donating region must still be preserved at the point where dilator capacity of the collateral-receiving region is exhausted.
2. The net collateral flow from the collateral-donating to the collateral-receiving must be reactive to a hyperemic stimulus, ie collateral resistance is not negligible.

Whereas these are absolute conditions, it has been a a matter of debate, whether additionally a stenosis in the collateral donor artery must be present for coronary steal to occur, as stated by Kern [69], Schaper [52], Becker [53] and Gould [63]. 

Schaper *et al*. showed in an ameroid-based chronic artery occlusion model in dogs that myocardial blood flow was non-homogenously distributed during vasodilation with lidoflazine [52]. The collateral-dependent area had a reduced coronary reserve in comparison to the normal myocardium and the pressure distal to the occlusion decreased markedly with administration of arterioral vasodilators. However he did not examine the influence of a stenotic lesion of the collateral donor artery on collateral steal.

In contrast, Becker focused exclusively on the experimental model with a stenosis in the collateral donor artery [53]. In response to hyperemia induced by dipyridamole, there was a decrease in blood flow in the collateral-dependent region below resting level. However, as there was no control group without a stenosis in the collateral donor artery, the conclusion could not be drawn that a contralateral stenosis was a necessary condition for coronary steal to occur.

Patterson and Kirk examined coronary steal in a one-vessel occlusion model in dogs [54]. Blood flow was determined during intracoronary adenosine infusion without and with an additional graded partial stenosis proximal to the origin of the collaterals. The magnitude of coronary steal increased with increments in the resistance, induced by the graded stenosis, but coronary steal was also present in the absence of a stenosis in the collateral donor artery. Other investigations have also demonstrated coronary steal in single vessel disease with intercoronary collaterals, obviating the condition of additional stenotic lesions in the supply artery [70, 71].

It becomes clear that the presence of a contralateral stenosis is a facilitating factor for the occurence coronary steal, but not a necessary condition. It is the interplay between the antegrade coronary flow reserve (if any), the flow reserve of the supply artery and the collateral conductance upon which the occurrence of steal depends [61]. Additionally, it is conceivable that for a region to be affected by coronary steal, the collateral flow must amount to a relevant proportion of the total flow for that region.

In a computer network model of myocardial steal, Demer *et al*. predicted the occurrence of steal already with a collateral conductance of >1% relative to normal maximal conductance (ie a CFI of 0.01) in a 60% proximal stenosis of the LCX supplying collaterals to an occluded LAD [62]. Assuming a higher collateral conductance of 20%, ie sufficient to provide normal resting perfusion, the necessary severity of the contralateral stenosis for coronary steal to occur was reached already at a cross-sectional area reduction of ≥ 20%. Furthermore, assuming that distal vasodilation would cause pressure to fall at the origin of the collaterals due to the normal anatomic tapering, Demer *et al*. concluded that coronary steal could theoretically occur in the absence of a stenosis in the supply artery [61]. This assumption was in accordance with observations by Harrison *et al*. [72]. 

The findings from experimental studies and derived simulations were confirmed in clinical studies.

Seiler *et al*. investigated the occurrence of coronary steal in response to intravenous adenosine in 100 patients with non-occlusive CAD [56]. A coronary flow velocity reserve of <1 obtained distal to the stenosis was defined as coronary steal and was observed in 10% (10 patients). Patients with steal showed superior collaterals compared with those without steal: CFI was 0.65±0.24 in patients with steal versus 0.29±0.18 in those without steal (p=0.0001) [56]. Furthermore, coronary steal was observed both with and without a stenosis in the contralateral vessel.

Elaborating upon the interplay of ipsilateral, contralateral and collateral resistances during hyperemia, Billinger *et al*. concluded that the coronary flow velocity reserve of a collateralized region could be more dependent on hyperemic vascular resistance changes of the collateral and collateral supplying area than on the ipsilateral stenosis severity [60]. 

Werner *et al*. studied 56 patients during recanalization of a CTO to assess the determinants of coronary steal [59]. It was concluded that coronary steal was mainly due to a hemodynamically significant donor artery lesion, but could also occur due to an impaired vasodilatory reserve of the microcirculation in the absence of a donor artery lesion.

#### Summary

In the setting of CAD, autoregulation of blood flow leads to uneven distribution of microvascular resistance between different coronary territories. In situations of increased myocardial oxygen demand, the accentuation of this constellation can lead to hemodynamic interactions between a collateral receiving and collateral supplying vascular bed in the form of coronary steal, defined as a drop of coronary blood flow below resting levels in the affected area. The minimal prerequisites for the occurrence of coronary steal are an exhausted dilator reserve in a collateralized region in the presence of preserved dilator reserve in the contralateral collateral donor region. Coronary steal is facilitated by a contralateral stenosis and good collateralization. The phenomenon of coronary steal is clinically relevant due to a prevalence of 10-20% in non-occlusive CAD and of a third to half of patients with a CTO.

### Redistribution of Blood Due to Altering Macrovascular Resistances

The presence of an epicardial stenosis increases macrovascular resistance and induces a pressure drop across the arterial network, which in turn presents the major growth trigger for intercoronary collaterals thus subjected to augmented fluid shear stress. In the course of several weeks, there is enlargement of preformed collaterals. When normal antegrade flow is restored by PCI, there is sudden normalization of the macrovascular resistance from high to negligible values. The impact of this sudden change in macrovascular resistance on collateral function can be scrutinized separately for the situation of chronic total occlusions (CTO) and non-occlusive stenoses.

#### Recanalization of CTO and Regression of Collateral Flow

Data from experimental studies have shown a regression of collaterals after release or reperfusion of a chronic total occlusion [73-75]. The decrease in collateral function is most likely explained by the redistribution of blood due to altering macrovascular resistances.

Werner *et al*. assessed collateral circulation in 21 patients with a CTO before coronary angioplasty and after recanalization by intracoronary Doppler flow velocimetry [76]. Basal collateral flow was determined before the first balloon inflation with two further collateral flow measurements after recanalization, one before the end of the procedure and one after 24 hours. CFI was 0.48±0.25 before the recanalization, significantly dropped to 0.21±0.16 after PTCA, but changed no further within 24 hours.

**Fig. (5):**
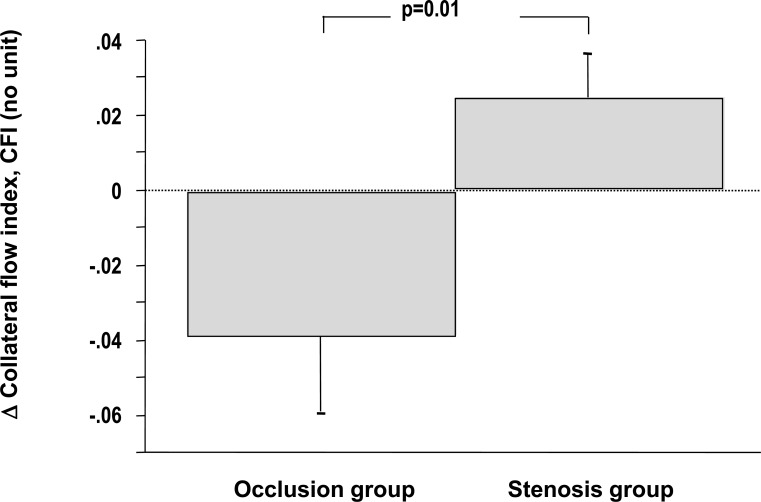
Changes in collateral flow between the first and the second balloon occlusion after revascularization in patients with (occlusion group) and without a chronic total occlusion (stenosis group). While patients with a prior total coronary occlusion show a trend for collateral de-recruitment, patients with a prior stenosis show intact collateral recruitment. See text for further explanation. [Data from Pohl *et al.* [77]].

Pohl *et al*. performed a similar study and additionally compared collateral flow changes determined at and after revascularization between 27 patients with a CTO (occlusion group) and 27 matched patients without total occlusion (stenosis group) [77]. Following revascularization, collateral flow index decreased in 17 of the patients in the occlusion group (63%) and in eight of the patients in the stenosis group (30%) (p = 0.03 between groups). Among patients with non-occlusive CAD collateral recruitment could be observed between the first and the second balloon inflation, while patients with a CTO showed a trend to collateral de-recruitment (see Fig. **5**).

With regard to the longer-term collateral function changes after recanalization exclusively in patients with a CTO, Werner *et al*. serially assessed collateral function in 103 patients [78]. In 93 patients without reocclusion (but 39 patients with restenosis) pressure-derived CFI decreased significantly from 0.39±0.12 at baseline to 0.30±0.13 after recanalization and a further significant decrease was noted after a mean follow-up of 5.0±1.3 months to 0.21±0.12. In 10 patients with reocclusion, CFI was not significantly different before recanalization at follow-up when compared to the CFI before recanalization at baseline.

**Fig. (6):**
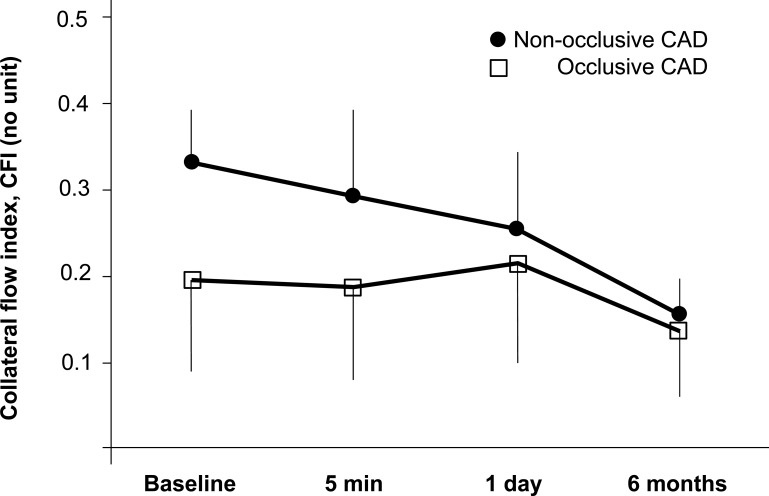
Behaviour of collateral blood flow before and after percutaneous coronary intervention for non-occlusive stenosis and chronic total occlusions. Patients with CTOs had a higher baseline CFI and a greater decline in CFI over time than those with non-occlusive lesions (p<0.0001) (error bars=1 SD). See text for further explanation. [Data from Perera *et al.* [79]].

Similarly, Perera *et al*. determined serial collateral flow measurements in patients with a CTO or high-grade lesions [79]. Pressure-derived CFI was determined during the initial PCI, as well as 5 minutes, 24 hours and 6 months after PCI (see Fig. **6**). At baseline, CFI was 0.23±0.10 with no significant changes 5 minutes and 24 hours after PCI. At 6 months however, CFI was found to have diminished significantly to 0.14±0.07 (p<0.001). Sufficient collateral flow, as defined by a CFI threshold of 0.25, was present in 47% of patients at baseline, but in only 4% at 6 months. Thus, functional collateral support following PCI declines but does not regress completely [79]. 

#### Revascularization of Stenotic Lesions and Risk of Restenosis

In the presence of a well-developed collateral supplying blood flow to a stenotic artery, it is conceivable that following PCI, the collateral perfusion pressure may compete with the restored antegrade perfusion pressure [80-83]. Consequently, the antegrade flow proximal to the origin of the well-developed collateral is reduced, thereby usually also affecting the part of the vessel treated by stent implantation.

As a coronary collateral can be seen as the natural counterpart to an artificially placed coronary bypass graft, the above described situation can be compared to a phenomenon encountered after coronary artery bypass grafting. In this situation, the effect of competitive flow introduced by an artificial bypass as opposed to a natural bypass can be observed.

It is well documented that accelerated progression of coronary lesions occurs in vessels which have been surgically bypassed [80, 81]. Even more relevantly in the context, Cashin *et al*. observed that the process of stenosis progression was ten times more frequent in bypassed arteries with only minimal atherosclerosis (defined as preoperative stenosis less than 50%) as in comparable arteries that were not bypassed [79]. These observations highlight the concept possibly also accounting for an increased risk of restenosis after stenting of a well-collateralized stenotic artery. Competitive flow by a natural bypass, ie a coronary collateral could predispose in a similar fashion to stent restenosis as competitive flow due to an artificial bypass does to progression of coronary lesions. 

The common mechanism possibly relates to the decreased shear stress on the endothelium resulting from the decreased antegrade flow. Shear stress is known to to be atheroprotective and, conversely, low flow and low shear stress, long known to critically affect the natural history of atherosclerosis, may also contribute to the development of restenosis after stenting of an atherosclerotic plaque [84]. Therefore, similar to predisposing to atherosclerosis [85, 86] the subsequent low(er) shear stress may also contribute to the development of stent restenosis [84, 87, 88]. 

Various studies have investigated the effect of the level of collateral flow on the risk for restenosis. The degree of collateralisation was estimated from visual assessment [89, 90] or determined by CFI [83, 91, 93] and based on data in the acute [88, 90, 91] or stable setting of CAD [83, 89, 92, 93]. Invariably, the number of patients in these studies were small and the findings rather inconsistent [91, 93]. 

Thus, Meier *et al*. in a recent publication, sought to determine the impact of the collateral circulation on the risk for restenosis in a metaanalysis [94]. 

**Fig. (7):**
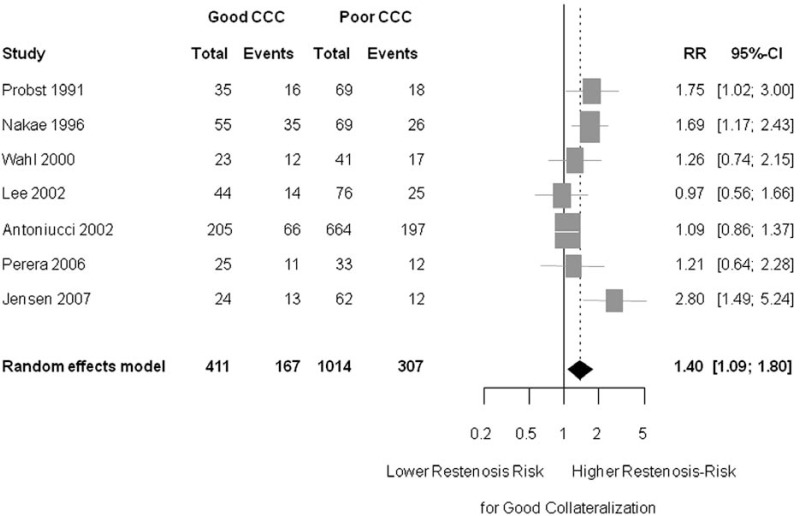
Forest plot of risk ratios (RR) for restenosis after percutaneous coronary intervention. Restenosis is defined as ≥ 50% diameter stenosis. Patients with good collateralization show a significantly increased risk for restenosis compared to patients with poor coronary collateralization. Markers represent point estimates of risk ratios, marker size represents study weight in random effects meta-analysis. Horizontal bars indicate 95% confidence intervals. [Data from Meier *et al.* [94]].

A total of 7 studies enrolling 1,425 subjects were integrated in this analysis. The assessment of collateralization was dichotomous, with poor collateralization defined as a Rentrop score ≤1 in the case of visual assessment [95] and CFI <0.25 in the case of intracoronary pressure measurements. The outcome was dichotomized as well in most studies, using a stenosis threshold of 50%. On average, across studies, the presence of a good collateralization was predictive for restenosis, with a risk ratio (RR) of 1.4 (95% CI 1.09 to 1.80); p = 0.009) (see Fig. **7**). The finding for an increased risk of restenosis with good collateralization was also consistent in the subgroup analyses for assessment with intracoronary pressure measurements or visual assessment. For the subgroup of patients with stable coronary artery disease, the RR for restenosis with ‘good collaterals’ was 1.64 (95% CI 1.14 to 2.35) compared to ‘poor collaterals’ (p = 0.008). For patients with acute myocardial infarction, however, the RR for restenosis with ‘good collateralization’ was only 1.23 (95% CI 0.89 to 1.69, p = 0.212) [94]. 

In conclusion, present data indicate an increased risk for restenosis in the presence of good coronary collateralization.

## STIMULI FOR LOWERING CORONARY COLLATERAL RESISTANCE

Apart from flow-mediated vasodilation, coronary collateral vasomotion is directly responsive to other stimuli. However, investigation of collateral vasomotion is complicated by the fact that collateral perfusion must transit not only the collateral vessels itself, but also the vessels up- and downstream of them [96]. Therefore, regulation of collateral blood flow is subject to control mechanisms not present in normally perfused myocardium [7]. Alterations in true collateral vascular tone have to be differentiated from those in the vasomotor tone of up- or downstream resistances, requiring special experimental preparations suitable only in the animal model.

### Autonomous Nervous System/ Neurohormonal Stimuli

#### α-Adrenergic Control

α_1_- and α_2_-adrenoreceptors mediate coronary vasoconstriction and thus oppose metabolic coronary vasodilation during exercise, but also limit coronary vasodilation in regions of ischemic myocardium [10]. 

Herzog *et al*. examined the effect of α_1_-blockade with prazosin on blood flow to a collateral-dependent myocardial region in dogs [97]. Myocardial blood flow was determined at rest and during treadmill exercise 9-14 days after acute occlusion of the left circumflex artery, resulting in a variable degree of subendocardial infarct in the collateralized region. During resting conditions, prazosin did not alter mean myocardial blood flow or the subendocardial-to-subepicardial flow ratio in either normally perfused or collateral-dependent myocardium. However, during exercise at comparable external workloads and comparable rate-pressure products, α_1_-blockade with prazosin lead to a significant increase of 27% in the normally perfused and to a significant 35% increase in the collateral-dependent myocardium compared with control. [97]. It was concluded that α_1_-adrenergic vasoconstriction inhibited coronary vasodilation during exercise, even in areas of collateral-dependent myocardium relatively early after coronary artery occlusion.

While the results of the above study would be consistent with a direct influence of α_1_-adrenergic activity on collateral function, other studies have refuted this hypothesis.

Bache *et al*. studied the ability of moderately well-developed coronary collateral vessels to undergo vasoconstriction in response to alpha-adrenergic stimulation and to ergonovine in intact dogs 4-16 weeks after collateral growth stimulation by LAD embolization [98]. To avoid possible β-adrenergic influences, the study was performed after administration of propranolol. Neither cardiac sympathetic nerve stimulation nor α1-adrenergic stimulation with phenylephrine or the selective α2-agonist B-HT 933 altered collateral flow. Ergonovine was found to act as a coronary vasoconstrictor principally by serotoninergic receptor activation and not by α-adrenoceptor stimulation.

Similarly, Hautamaa *et al*., also in dogs 4-16 weeeks after ameroid-based LAD occlusion, examined the ability of moderately well-developed coronary collateral vasculature to undergo vasoconstriction in response to alpha-adrenergic agonists, vasopressin and angiotensin, and vasodilation in response to nitroglycerin [99]. Neither the selective α1-adrenergic agonist phenylephrine nor the α2-agonist B-HT 933 decreased retrograde flow.

Harrison *et al*. studied 13 dogs, 6-10 months after ameroid occlusion of the left circumflex artery to determine, if mature coronary collateral vascular smooth muscle contained functioning alpha-adrenergic receptors [100]. Regional myocardial blood flow was measured with the microsphere method with aortic pressure held constant. Vasodilation with adenosine was induced and normal zone and transcollateral resistance was calculated during infusion of the α-adrenergic agonist methoxamine or the α_2_-adrenergic agonist clonidine. Normal zone resistance increased both in response to methoxamine and clonidin, whereas transcollateral resistance was unchanged. However, clonidine caused a 22% decrease in collateral flow, which was mediated by vasoconstriction of resistance vessels in the collateral-dependent region. Furthermore, the left anterior descending artery and mature collaterals were examined in organ baths. The α1-adrenergic agonist phenylephrine and clonidine-induced constrictor responses in the LAD but not in mature collaterals. It was concluded that mature coronary collaterals did not contain functioning alpha-adrenergic receptors [100]. 

In conclusion, coronary collaterals are not directly responsive to α-adrenergic influences. The observed changes in collateral flow in response to α-adrenergic influences are due to alteration of the resistance in non-collateral vessels, as transcollateral resistance was shown to remain unchanged. In the presence of α-adrenergic blockade, the vasomotor tone in the resistance vessels of the collateral-dependent myocardium is abolished, leading to increased collateral flow. Conversely, α-adrenergic stimulation enhances the vasomotor tone and thus reduces collateral flow. α-adrenergic nervous activity does, however, not have a direct influence on collateral vasomotor function per se.

#### β-Adrenergic Control

While mature collateral vessels have been found to be devoid of functioning α-receptors, Feldman *et al*. demonstrated beta-adrenergic-mediated relaxation with affinities for both agonists and antagonists, compatible with a mixed population of β1- and β2-adrenergic receptors in collateral arteries developed after placement of ameroid constrictors in dogs [29]. 

Herzog *et al*. assessed the effect of beta-adrenergic blockade with timolol on myocardial blood flow during rest and graded treadmill exercise in dogs with a collateral-dependent myocardial region containing infarct [101]. At comparable rate-pressure products during exercise, total myocardial blood flow was 24% lower after timolol. Furthermore, timolol caused a decrease of subepicardial blood flow in the collateral region comparable to that in the normally perfused region. However, the effect of beta-adrenergic blockade could be attributed to either the vasoconstriction of the collateral vessels or the resistance vessels in the collateral zone.

Elaborating on this, Traverse *et al*. observed an increase of transcollateral resistance, but also of small-vessel resistance in response to beta-adrenergic blockade with propranolol during exercise in collateralized dogs without infarction [31]. Collateral zone blood flow consequently decreased.

In clinical studies, the effect of the autonomous nervous system on the vasomotor response of collaterals used the cold pressor test, known to strongly activate the adrenergic nervous system [102]. While the study by Uren *et al*. [103] measuring regional myocardial blood flow with PET in 9 patients with collateral-dependent myocardium, produced conflicting results, the study by de Marchi *et al*. reported results in favour of a beta-adrenergic mediated coronary collateral vasodilation in response to the cold pressor test [104]. In 30 patients with chronic stable CAD, two consecutive randomized occlusive collateral flow measurements were performed, one with, the other without a preceding 3-minute cold pressor test. Collateral flow reserve induced by the cold pressure test was 1.18. However, in the subgroup of patients on beta blocker treatment, CFI was not additionally augmented in response to the cold pressor test. It was concluded that this finding might reflect coronary collateral vasodilation mediated by the sympathetic nervous system.

In summary, the data from experimental studies are consistent with both the existence and functionality of beta-adrenergic receptors on coronary collateral vessels. The decrease in collateral flow in response to beta-blockade is due to changes both in collateral and small-vessel resistances. The augmented collateral function in response to sympathetic stimulation is abolished by beta-adrenergic blockade.

### Acute Single and Repetitive Myocardial Ischemia Episodes

#### Acute Single Ischemia Episode

It has been demonstrated in experimental studies that collateral blood flow increases during the first hour [105] and even 24 hours [106] after acute coronary occlusion. The studies could however not determine, if the progressive increase in collateral blood flow could be attributed to changes in the vascular resistance in the ischemic area, direct collateral vasodilator responses or changes in extravascular forces.

In this context, Lamping *et al*. directly visualized epicardial microvessels and demonstrated that native collateral vessels in dogs dilate in response to an acute coronary occlusion for at least 15 minutes after the occlusion [107]. Furthermore, the dilation in the native collateral vessels was shown to be mediated by ATP-sensitive K+ channels and this response was also replicated in stimulated collaterals [108] and with selective activation of ATP-sensitive K+ channels by aprikalim [109]. 

#### Repetitive Ischemia

Repetitive occlusions have routinely been employed to induce collateral growth before definitive coronary occlusion in the chronic ischemia model. Developing ischemia tolerance to repetitive occlusions has, however, been observed already in the short term, before the start of collateral growth. This observation was attributed to either ischemic preconditioning or collateral recruitment.

Fujita *et al*. determined collateral blood flow and regional myocardial function during multiple one-minute coronary occlusion in dogs [110]. After sixteen one-minute coronary occlusions collateral blood flow velocity nearly tripled from 0.7±0.1 to 2.0±0.2 cm/sec and systolic segment shortening in the collateral-dependent zone improved slightly.

Yamanishi *et al*. investigated the effect of different occlusion protocols on collateral blood flow and regional myocardial function [111]. Three occlusion protocols were performed consecutively, with total occlusion time amounting to 5 minutes in each protocol. The first protocol comprised thirty occlusions of 10 seconds, the second protocol five occlusions of 1 minute and the third protocol 1 occlusion of 5 minutes. Determined in a test occlusion and in a separate occlusion, 1 minute after the end of the respective protocol, collateral blood flow was measured as a stepwise decrease in LCX flow on release of the LAD occlusion. Collateral blood flow in the first test occlusion was 1.6 ml/min, remained unchanged after the first protocol, but increased to 3.5 ml/min after the second protocol and to 3.5 ml/min after the third protocol.

These experimental studies show that collateral blood flow increases in response to repetitive occlusions, but missing electrocardiographic data do not document a potential decrease in myocardial ischemia in response to repetitive ischemia.

Sakata *et al*. aimed to investigate the interaction between ischemic preconditioning and collateral recruitment in 18 patients undergoing elective PTCA of the left anterior descending artery [112]. Patients were subdivided according to recruitable or non-recruitable collaterals as determined by myocardial contrast echocardiography (MCE). Essentially, the collateral blood flow in the patient group with recruitable collaterals practically prevented surface ECG signs of ischemia during all coronary balloon occlusion, while patients without recruitable collaterals had a significant decrease in ST segment shift from the first to the third balloon occlusion. The crude assessment of collateral flow did not allow to attribute decreases in ST segment shift to collateral recruitment or ischemic preconditioning. Furthermore, ischemia was quantified only by surface ECG, a method unsuitable for the both the detection and grading of slight ischemia.

Billinger *et al*. assessed the contribution of ischemic as well as adenosine-induced preconditioning and of collateral recruitment to the development of tolerance against repetitive ischemia [113]. Collateral blood flow was quantitatively determined by pressure-derived CFI and ischemia was quantified by the ST segment elevation in intracoronary ECG during three subsequent 2-min balloon occlusions. Furthermore, the 30 patients were divided into two groups according to the pretreatment with intracoronary adenosine (2.4 mg/min for 10 min starting 20 min before the first occlusion, n = 15) or with normal saline (control group, n = 15).

**Fig. (8):**
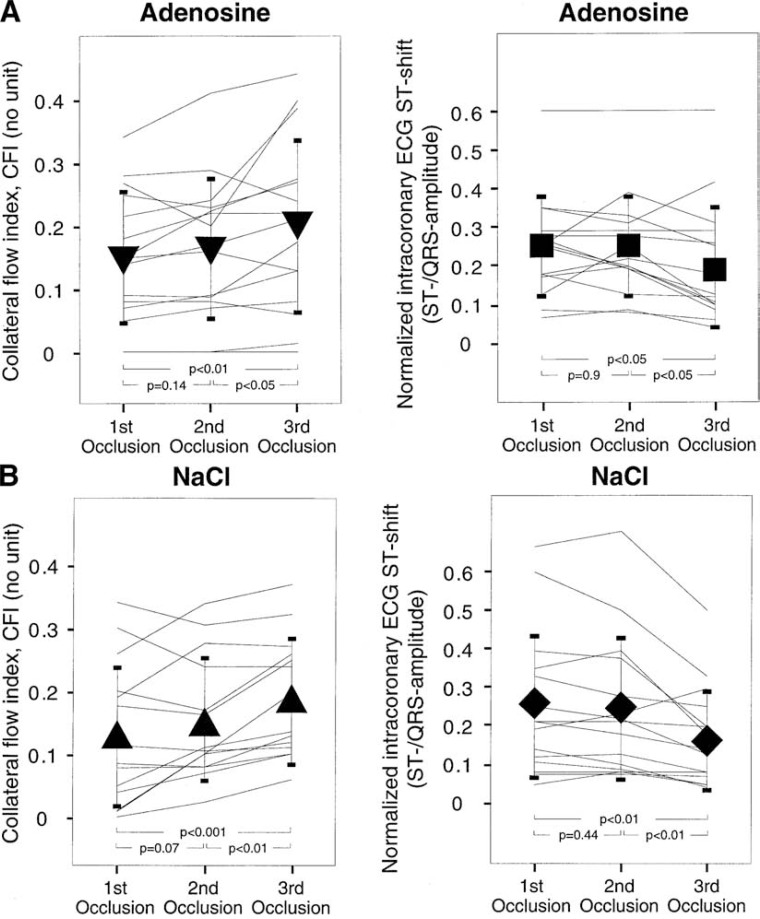
Intraindividual changes in collateral flow (left panels) and in normalized ECG ST shift in the intracoronary ECG (right panels) during three consecutive balloon occlusion of each 2 minutes duration. Upper panels show data for the group pretreated with adenosine infusion, lower panels shows control group without adenosine pretreatment (NaCl infusion). Collateral flow shows a trend for an increase from the first to the second occlusion and a significant increase in the third balloon occlusion. ST shift in the intracoronary ECG is significantly lower only in the third balloon occlusion. See text for further explanation. [Data from Billinger *et al.* [113]].

Collateral flow index at the first occlusion was not different between the groups (0.15 ± 0.10 in the adenosine group and 0.13 ± 0.11 in the control group, p = NS), and it increased significantly and similarly to 0.20 ± 0.14 and to 0.19 ± 0.10, respectively (p < 0.01) during the third occlusion (see Fig. **8**). The intracoronary ECG ST elevation (normalized for the QRS amplitude) was not different between the two groups at the first occlusion (0.25 ± 0.13 in the adenosine group, 0.25 ± 0.19 in the control group). It decreased significantly during subsequent coronary occlusions to 0.20 ± 0.15 and to 0.17 ± 0.13, respectively. There was a correlation between the change in collateral function from the first to the third occlusion and the respective ECG ST elevation shift (r^2^ = 0.29, p = 0.002). However, collateral recruitment accounted for only 30% of the observed variation in ECG ST segment shift during recurring episodes of ischemia, indicating that ischemic preconditioning likely was also a factor contributing to ischemic tolerance.

#### Summary

Collateral flow is able to increase during ischemia, as well as in response to repetitive ischemia episodes. The increase in flow is at least partly attributable to collateral vasodilation. Furthermore, it is likely that ischemic preconditioning, apart from collateral recruitment, is a factor contributing to myocardial tolerance to ischemia.

### Pharmacological Stimuli

**Nitrovasodilators.** Nitrovasodilators, such as nitroglycerin preferentially dilate arterial vessel >100 μm. [114, 115]. With regard to the coronary collateral circulation, several studies in collateralized dogs have demonstrated intact endothelial-dependent vasodilator mechanisms in response to the NO-dependent agonists bradykinin and acetylcholine, as well as the endothelium-independent nitroglycerin [116, 117]. Collateral flow was shown to increase in response to intracoronary nitroglycerin and isosorbide dinitrate in dogs in the early (2-4 weeks), as well as in the later stages of collateral development (8-14 weeks) following embolisation of the left anterior descending artery [118, 119]. Conversely, collateral blood flow was decreased by inhibition of NO synthesis [120, 121], and it was shown that this was caused directly by an increase in collateral resistance, thus proving a direct influence on collateral vasomotion [122]. Furthermore, Frank *et al*. showed that nitric oxide contributed to maintenance of collateral vasodilation by exerting a substantial tonic dilating effect on coronary collateral vessels in collateralized dogs at rest [122]. 

During exercise, Traverse *et al*. showed that inhibition of NO synthesis decreased collateral blood flow and that this was predominantly due to a near doubling of collateral resistance, ie a direct effect on collaterals [123]. Administration of nitroglycerin during exercise did however not lead to an increase in collateral flow to a collateral-dependent myocardial region in dogs, consistent with the notion that the endogenous NO system is already maximally recruited during exercise in the collateral-dependent region [124, 118, 125]. In support of this hypothesis is the observation of Klassen *et al*. that collateral blood flow during exercise in dogs with permanent occlusion of the LAD decreased upon inhibition of NO production, but subsequent infusion of nitroglycerin improved collateral zone blood flow [126]. 

Clinical studies on the influences of NO on collateral vasomotion have focused on the enhancement of NO in the form of nitroglycerin.

Goldstein *et al*. measured collateral flow using the retrograde flow method in patients with advanced coronary artery disease undergoing saphenous vein bypass [127]. Collateral resistance decreased during intraaortic infusion of nitroglycerin and collateral flow increased. Furthermore, responsiveness to nitroglycerin, as evaluated by percent reduction in coronary collateral resistance, was not correlated with angiographic appearance of collaterals.

Feldman *et al*. measured collateral blood flow during transient balloon occlusion in the left anterior descending artery in 21 patients with CAD [128]. Measurements were made before and during administration of intravenous nitroglycerin. A calculated coronary collateral resistance index decreased in response to intravenous nitroglycerin in 13 of 21 patients and responsiveness did not appear to depend on the presence or absence of angiographically visible collateral vessels [128]. 

In contrast, Piek *et al*. found that nitroglycerin decreased collateral resistance and increased collateral flow only in the presence of spontaneously visible collateral vessels [37]. Of note, nitroglycerin was given as an intracoronary bolus to avoid systemic effects possibly having an indirect effect on collateral flow. However, the intracoronary route also lead to a systematically higher dose in the group with spontaneously visible collaterals, thus setting the group with only recruitable collaterals at a significant disadvantage.

**Adenosine**. In contrast to vasodilators, adenosine is a potent vasodilator of coronary arterioles <100 μm, [27, 28] but only a weak dilator of arterial vessels >100 μm. While clinical studies have used adenosine to study its influence on collateral function (see above), in the experimental setting also acadesine was used. Acadesine is an adenosine-regulating agent pharmacologically silent in normal circumstances, but augments adenosine production in metabolically stressed tissues such as in ischemia [129]. Systemic hemodynamic changes are thus avoided. Ishibashi *et al*. examined the effect of acadesine on blood flow to collateral-dependent myocardium during exercise in collateralized dogs and a control group [130]. Collateral growth was induced by the repetitive coronary occlusion technique and was followed by permanent occlusion. After 5-7 days of permanent occlusion blood flow measurements were determined during control exercise and exercise after acadesine infusion. In dogs with a collateral-dependent myocardial region, acadesine increased blood flow by 24±5% during exercise. The increase in collateral zone blood flow in response to acadesine resulted from a 25% decrease in transcollateral resistance and a 20% decrease in small-vessel resistance in the collateral-dependent myocardium [130]. 

## EXTRACORONARY PHYSICAL DETERMINANTS OF COLLATERAL FLOW

Apart from vascular properties of both coronary and collateral arteries, extravascular determinants are also of importance concerning the collateral circulation. The salient features will subsequently be discussed.

### LV Preload

Ischemia precipitates left ventricular dysfunction und ultimately leads to increases in left ventricular preload, characterized by the left ventricular end-diastolic pressure (LVEDP) [131, 132]. Coronary collaterals reduce or even abolish ischemia and might therefore prevent or attenuate the potentially deleterious effects of LVEDP rises on myocardial perfusion. Conversely, LVEDP might negatively influence the mostly diastolic collateral flow via the transmission of LV cavity pressure tending to collapse the collateral vessels [133, 134]. Yet, it could be imagined that the hemodynamic cross-talk between the LV cavity and the epicardial vasculature might also be of the inverse relationship, ie higher LVEDP in the presence of higher collateral flow.

In this context, de Marchi *et al*. obtained hemodynamic parameters in 50 patients with CAD at rest and during a brief coronary occlusion [135]. The appearance of ECG signs of ischemia during coronary occlusion defined insufficient collaterals, whereas the absence defined sufficient collaterals. LVEDP was higher during coronary occlusion in the group with insufficient collaterals and increased steadily during the occlusion. In the group with sufficient collaterals, LVEDP increased only initially to reach a plateau towards the end of the occlusion. Beyond an occlusive LVEDP of >27 mmHg, collateral and peripheral resistance increased as a function of LVEDP, in agreement with data from experimental studies, predicting a Waterfall effect, ie collapse of coronary collaterals at a threshold of ~25-30mmHg [134, 136, 137]. 

In a similar study with 25 patients with CAD, Hoole *et al*. confirmed the results by de Marchi *et al*. [138]. LVEDP increased in patients with lower pressure-derived CFI, but stayed constant in patients with adequate collaterals. LV diastolic dysfunction following coronary occlusion was inversely correlated with CFI. Furthermore, patients with poorer collaterals had lower LVEDP at baseline (r = 0.33, p = 0.02), consistent with the notion, that baseline LVEDP may be high in the patients with good collaterals due to a direct and constant scaffold effect of collateral vessels on the LV cavity, as proposed by de Marchi *et al*. [135, 139]. 

### Heart Rate

Shortening of diastole during increased heart rates may influence collateral flow, which occurs almost exclusively during diastole. In experimental studies, tachycardia induced by atrial pacing decreased collateral blood flow to an entirely collateralized LAD region [140], whereas after acute coronary artery ligation in dogs, overall flow in the ischemic myocardium did not change significantly during atrial pacing, but a redistribution from the subendocardial to the subepicardial region was observed [141]. 

Fedor *et al*. compared the effects of exercise- and pacing induced tachycardia on collateral flow in dogs 11-12 weeks after ameroid-based occlusion [17]. Collateral flow increased during both pacing and exercise, but there was a subendocardial perfusion deficit. Furthermore, blood flow to the collateral-dependent region was higher during exercise than during pacing, indicating a differential effect on collateral function of exercise- vs pacing-induced tachycardia.

### LV Contraction

Coronary flow is impeded in systole due to cardiac contraction and contractility is the main determinant of systolic flow impediment [142]. Myocardial compressive forces equal intrathoracic pressure at the epicardium and increase towards the endocardial surface, creating a gradient of tissue pressure [143, 144]. The intravascular distending pressure counteracts, with the net result that systolic flow is inhibited in the subendocardium and directed toward the subepicardium.

With regard to the influence of LV contraction on the collateral flow, Russell *et al*. conducted a study in dogs with a myocardial area entirely supplied by collateral vessels [145]. Compared with the normally perfused region, collateral blood flow was more inhibited at corresponding layers of the myocardium and inhibition was actually magnified at the mid wall.

### LV Afterload

The predominant effect of LV contractility over left ventricular pressure on the systolic impediment of coronary flow [142] is consistent with the notion that changes in afterload, characterized by wall stress at peak systole, will not relevantly alter coronary blood flow in general or collateral flow in particular.

Brown *et al*. specifically investigated the effect of afterload changes on collateral flow [140]. Variation of mean systolic pressure by application of intraaortic counterpulsation selectively during systole had very little effect on the collateral flow to the chronically occluded LAD region, when diastolic pressure was kept constant.

### Summary

This pathophysiological review presents the salient features relating to the functional adaptation of coronary collaterals.

It has been shown that collaterals functionally adapt to flow-mediated dilatation induced by the concerted effect of exercise or by pharmacologic stress with adenosine. The hereby observed heterogenous responses produced by vascular adaptations in both coronary collateral and coronary non-collateral vessels have then lead to examine the consequences of a heterogeneous vascular resistance distribution on coronary blood flow.

Altered *microvascular* resistance distribution forms the basis for coronary steal, the occurrence of which is facilitated by well-developed collaterals and the presence of a contralateral stenosis. Changes in the *macrovascular* resistance distribution induced by restoration of antegrade blood flow by PCI lead to the decline of collateral support but not to complete regression. Furthermore, present data indicate an increased risk for restenosis in the presence of good coronary collateralization.

The responsiveness of coronary collaterals to stimuli other than flow-mediated vasodilation were examined predominantly in the experimental setting. While α-adrenergic nervous activity does not have a direct influence on collateral vasomotor function, collaterals are reactive to beta-adrenergic activity. Both single and repetitive ischemia episodes lead to functional adaptation of collaterals. Pharmacological stimulation with nitrovasodilators or with exogenous adenosine or adenosine-like acting agents has a direct influence on collateral vasomotion.

Last, extracoronary determinants of collateral function were discussed. LV preload negatively affects collateral function beyond a LVEDP of >27 mmHg, while it appears to be unaffected by isolated changes in LV afterload. LV contraction is the main determinant of systolic flow impediment and collateral blood flow is more inhibited at corresponding layers of the myocardium compared with a normally perfused region. Increases in heart rate shorten diastole and thus the time frame, in which collateral blood flow almost exclusively occurs. Depending on the degree of collateral maturity, partly limited capacity to compensate for the shortened perfusion window and the increased demand is exhibited.

In summary, this review has shown that the collateral circulation is capable of functional adaptation, with direct collateral vasomotion being an integral part. The conception of coronary collaterals as rigid tubes is obsolete.

## Figures and Tables

**Fig. (9):**
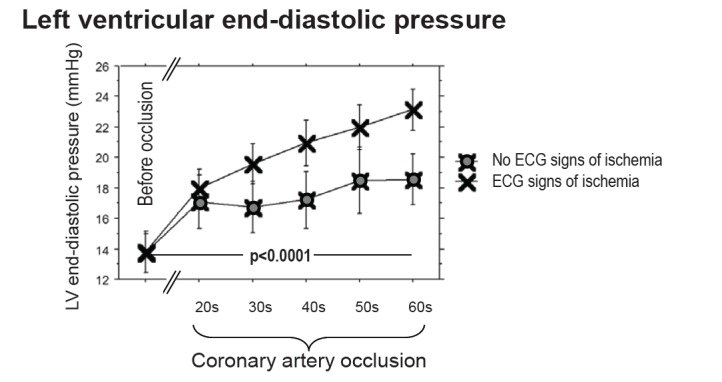
Intraindividual LVEDP changes during coronary balloon occlusion in patients with sufficient (blue symbols) and insufficient collaterals (black symbols). LVEDP raises similarly at the start of the coronary occlusion in both groups, but reaches a plateau in patients with sufficient collaterals, whereas LVEDP in patients with insufficient collaterals continues to increase. See text for further explanation. [Data from de Marchi *et al.* [135]].
